# Nursing care for the Warao people: an experience report based on transcultural theory

**DOI:** 10.1590/1980-220X-REEUSP-2023-0035en

**Published:** 2024-01-08

**Authors:** Ana Flávia Silva Lima, Cayo Emmanuel Barboza Santos, Nemório Rodrigues Alves, Mário César Ferreira Lima, Jorgina Sales Jorge, Heloísa Wanessa Araújo Tigre, Ana Valéria Alves de Almeida, Tatiane da Silva Santos, Laís de Miranda Crispim Costa

**Affiliations:** 1 Universidade Federal de Alagoas, Maceió, AL, Brazil.; 2 Secretaria Municipal de Saúde, Maceió, AL, Brazil.

**Keywords:** Nursing, Nursing Theory, Primary Health Care, Indigenous Peoples, Ceremonial Behavior, Enfermería, Teoría de Enfermería, Atención Primaria de Salud, Pueblos Indígenas, Conducta Ceremonial, Enfermagem, Teoria de Enfermagem, Atenção Primária à Saúde, Povos Indígenas, Comportamento Ritualístico

## Abstract

**Objective:**

To report on the experience of nurses from the Street Clinic in caring for the Indigenous Venezuelan population of the Warao ethnic group in Maceió/AL based on Madeleine Leininger’s Transcultural Theory.

**Metodology:**

A descriptive study, of the experience report type, based on the care of the Warao Indigenous population in the light of Madeleine Leininger’s Transcultural Theory, carried out during the year 2022.

**Results:**

Light technologies were used to form bonds and understand the cultural universe of the Warao people. The concepts of preservation, accommodation and cultural restructuring of care from Leininger’s transcultural theory helped to elucidate the practice. Comprehensive care was offered in accordance with the programs recommended by the Ministry of Health, with transcultural care, including respect for refusal of care. The language barrier and health beliefs represented challenges in the context of singular care.

**Final considerations:**

The experience of nurses from the Street Clinic in caring for the Indigenous population favored significant social interaction and expanded the possibilities for achieving comprehensive health care. The application of Transcultural Theory proved to be an effective and congruent device for health care.

## RESUMO

**Objetivo:**

Relatar a experiência de enfermeiros do Consultório na Rua no cuidado à população indígena venezuelana da etnia Warao em Maceió/AL a partir da Teoria Transcultural de Madeleine Leininger.

**Metodologia:**

Estudo descritivo, do tipo relato de experiência, a partir do cuidado da população indígena Warao à luz da Teoria Transcultural de Madeleine Leininger, realizado durante o ano de 2022.

**Resultados:**

Utilizaram-se tecnologias leves para formação de vínculo e compreensão do universo cultural do povo Warao. Os conceitos de preservação, acomodação e reestruturação cultural do cuidado da teoria Transcultural de Leininger contribuíram para elucidar a prática vivenciada. Ofertou-se uma atenção integral conforme os programas preconizados pelo Ministério da Saúde, com cuidado transcultural, inclusive com respeito à recusa de assistência. A barreira linguística e as crenças no que tange à saúde representaram desafios no contexto do cuidado singular.

**Conclusão:**

A experiência de enfermeiros do Consultório na Rua no cuidado a população indígena favoreceu uma significativa interação social e ampliou as possibilidades para o alcance da integralidade em saúde. A aplicação da Teoria Transcultural se mostrou como um dispositivo eficaz e congruente para o cuidado em saúde.

## RESUMEN

**Objetivo:**

Referir la experiencia de enfermeros de una Unidad Sanitaria Móvil en la atención a la población indígena venezolana de la etnia Warao, en Maceió/AL, a partir de la Teoría Transcultural de Madeleine Leininger.

**Metodología:**

Se trata de un estudio descriptivo, del tipo informe de experiencia, basado en la atención a la población indígena Warao a la luz de la Teoría Transcultural de Madeleine Leininger, realizado durante el año 2022.

**Resultados:**

Se utilizaron tecnologías leves para crear vínculos y comprender el universo cultural del pueblo Warao. Los conceptos de preservación, acomodación y reestructuración cultural de los cuidados de la teoría transcultural de Leininger ayudaron a dilucidar la práctica. Se ofreció atención integral según los programas recomendados por el Ministerio de Salud, con atención transcultural y se respetó el rechazo a la atención. La barrera lingüística y las creencias sanitarias representaron desafíos en el contexto de la atención singular.

**Conclusión:**

La experiencia de los enfermeros de la Unidad Sanitaria Móvil en la atención a la población indígena favoreció mucho la interacción social y amplió las posibilidades de lograr una atención sanitaria integral. La aplicación de la Teoría Transcultural demostró ser un dispositivo eficaz y adecuado para el cuidado de la salud.

## INTRODUCTION

There are an estimated 400 million Indigenous people in the world, with 764,000 Indians living in Brazil. They are distributed throughout the national territory in approximately 305 peoples with 180 different dialects^(1)^. From a historical perspective, the Indigenous population has been marked since the period of colonization by mistreatment, slavery, sedentarization, wars, genocide and epidemics^(1,2)^. This group still suffers from tensions, threats and vulnerabilities, with weakened health determinants and conditions related to poor hygiene and nutrition, overcrowded housing, environmental contamination and infections^(1-3)^.

Given the diversity of Indigenous groups, each has its own form of socio-political, economic and related organization in dealing with the health-disease process^(1)^. In this context, Brazil has been the scene of Venezuelan migration, driven above all by the country’s political and economic crisis. Among the immigrants are the Indigenous peoples of the Warao ethnic group^(4)^, who are traditionally inhabitants of the Orinoco River delta, made up of a very diverse ethnic group in terms of their forms of social organization and customs, sharing a common language, also homonymously called Warao, and Spanish at varying levels of fluency^(4,5)^. Spiritual rituals occupy a central place in the process of illness and healing for these Indigenous people. When a member of the ethnic group falls ill, the tradition is that the first diagnosis should come from one of their shamans and that the person can only be referred for conventional treatment after their release^(5)^.

Thus, it is known that traditional Indigenous medicine has been ratified internationally as a growing part of health care systems. Its understanding in the modern world constitutes a scenario of therapeutic plurality and epistemological diversity, in which health practices have a place^(6)^. In this context, there is growing advocacy for the integration of traditional medicine with the body of knowledge and services of conventional medicine, but it is known that the actions of current public policies aimed at Indigenous health promote a limited exchange, rather than integration, as established in the national Indigenous policy^(6,7)^.

Among the main nursing theories that emphasize the nature and phenomenon of care is the Transcultural Theory, proposed by nurse Madeleine Leininger^(8)^. This theory is considered comprehensive and welcoming, as it encompasses and understands health demands in multicultural populations and communities^(9)^, making nursing capable of (re)signifying the diversities, culture and also the common elements of individuals in their socio-cultural contexts as determining characteristics of their state of health or illness, as well as providing directions for an attentive and respectful view of the subjects’ behavior^(10)^.

Therefore, nursing work in Indigenous health requires an intercultural approach, based on the implementation of humanized care that respects the beliefs and values of the assisted group. It is worth considering that during the systematization of Nursing care, care guided by a Nursing theory is essential^(11)^, thus Madeleine Leininger’s Transcultural Theory represents an appropriate theoretical support in caring for the Warao Indigenous community.

In view of the above, the Street Clinic is a gateway to health services for the homeless population and people in conditions of vulnerability, working with comprehensive actions to meet the needs of this group^(12)^. It’s worth mentioning that when the Warao people arrived in Brazil, many were homeless, having to beg for money and food to survive, and were later taken into shelters^(4,5)^, thus also becoming a target audience for the Street Clinic.

In this context, considering the challenge faced by the Street Clinic teams in Maceio/AL to provide comprehensive care to a foreign ethnic group with its specificities and the history of growing Venezuelan Indigenous immigration to Brazil, it is important and relevant to discuss the subject for nursing and the health field. Thus, in order to add more scientific production on the subject, this paper aims to report on the experience of nurses from the Maceió/AL Street Clinic in caring for the Indigenous population in the light of Madeleine Leininger’s Transcultural Theory as a theoretical reference for practice.

## METHOD

### Study Design

This is a descriptive study of nurses’ experiences in caring for the Warao Indigenous population in the light of Madeleine Leininger’s Transcultural Theory. This type of article describes an experience that can make a relevant contribution to a particular area of activity. It should be written in a contextualized and objective way, contributing by stimulating exchanges and propositions of ideas in the field in question. Its purpose is to socialize an experience, spark debate and enable reflection^(13)^. In the meantime, the Transcultural Theory has guided nursing care based on the three forms of action that the theory emphasizes: cultural preservation of care, cultural accommodation of care and cultural restructuring of care^(14)^.

The concept of cultural preservation of care can be understood as providing assistance in order to facilitate and enable the user to maintain and preserve culturally based healthy habits. On the other hand, the cultural accommodation of care refers to revealing ways of negotiating and adapting people’s health and life habits according to their beliefs. And the cultural restructuring of care is the new construction of health and life in a way that is meaningful and congruent for the individual^(8,14)^.

In addition, this study has a cross-sectional time frame and for the purposes of describing this experience, the period from February to December 2022 was considered, in the exercise of nursing care in the work of the Street Clinic in Maceió/AL.

### Setting

Nursing care took place in some of the settings where the Maceio Street Clinic teams operate, such as the street environment, since some Indigenous people were in these conditions, and in shelters and makeshift houses. During the winter, in June 2022, the funds received from the federal government, together with the heavy rains, led to the organization of a shelter for Indigenous immigrant families in the city center. This shelter has ten rooms to accommodate the families, as well as a structure with bathrooms, an activity room, a kitchen, a cafeteria and a laundry room. Other shelters were organized later, but the structure of these places is precarious, so that each family is accommodated in a single room, i.e. one room per family, regardless of their composition.

It is worth mentioning that in the state of Alagoas, only the city of Maceio has Street Clinic teams, with six multidisciplinary teams that work on the move and in conjunction with teams from the territory’s Primary Health Care Center in order to provide assistance to the homeless population or those in similar conditions in all of the capital’s health districts during the three shifts. The teams coordinate with the Health Care Network to guarantee comprehensive care at the various levels of complexity^(15,16)^.

### Participants

Nursing care was provided to the Warao people by nurses from the Street Clinic and their respective teams, as well as two master’s degree students from the Federal University of Alagoas’ Postgraduate Nursing Program, as part of the production of information for their research. It’s worth noting that the Street Clinic teams in Maceio are qualified by the Ministry of Health under modality II, which involves the inclusion of at least six professionals, three of whom must be university graduates and three of whom must be high school graduates^(15,16)^. Although the organization, management and attributions of the Street Clinic are similar in the other states of Brazil, it is necessary to consider the regional particularities that singularize health care.

### Ethical Aspects

This study was developed through the experience of nurses who work in the Street Clinic in Maceio/AL, caring for Indigenous people of the Warao ethnic group, immigrants from Venezuela. Therefore, it deals with the description of lived practice, challenges in nursing care and care strategies based on the theoretical support of Madeleine Leininger’s Transcultural Theory. As such, no information was produced that could identify people or institutions, and there was no need to submit it to Research Ethics Committee.

## RESULTS

In addition to serving the homeless population, the Street Clinic teams are also a reference in caring for Indigenous immigrants from Venezuela in Maceió/AL. The teams’ first contact with the Warao Indigenous people was in February 2022, with a group of around 20 people. Over the following months, the team got closer and built bonds, expanding the range of people and by December 2022, according to the registrations made, there were around 100 Warao Indigenous people.

In this context, the Street Clinic teams are part of Primary Health Care and must therefore follow the guidelines and orientations of the public policies recommended by the Ministry of Health^(15)^. In assisting the Warao people, we sought to offer comprehensive care with respect for their culture and beliefs, based on Madeleine Leininger’s Transcultural Theory. To do this, the team had to immerse themselves in their care practices and worldview, which allowed them to build a relationship of trust anchored in the bond and welcome that is essential for health care. In this way, the team was able to facilitate entry into the health services, building bridges with the care networks, while monitoring the user through referrals and consultations, taking into account the preservation of cultural care related to religious rituals associated with health, but with accommodation and restructuring of cultural care through negotiation, adjustment and reconstruction, which was achieved through the interpersonal relationship built. Meetings were also organized to make the interests and needs of this public viable. In addition, there were nursing consultations; health education activities; nursing procedures; interpretation of laboratory tests; rapid tests for Sexually Transmitted Infections (STIs); as well as meeting the needs presented at the time of the approach.

In order to carry out these actions, the nurses, together with the multidisciplinary team, were faced with the need for an understanding of the cultural differences and forms of organization of the Warao people. As already mentioned, this population lived on the streets, in shelters, or in precarious housing that was too small for the number of family members. In this situation, prenatal consultations were carried out in the office team’s van [work car] because it was impossible to do them in the place where they lived. These situations, which required (re)adaptation, made it possible for the professionals to reflect, grow and learn from reinventing the way they do health care in order to provide comprehensive care that respects cultural values.

In addition, habits and forms of domestic organization were observed that offer risks and can compromise health, causing other problems for an already fragile population: such as the arrangement of pots, plates and utensils on the floor of houses and bedrooms and the habit of disposing of human and food waste in inappropriate places, close to the group’s living spaces. Childcare appointments revealed the presence of cavities and poor oral hygiene.

It is worth mentioning that the Warao people, in their place of origin, live in houses near rivers, with minimal furniture and no basic sanitation. It was therefore initially a challenge for the health team to understand these cultural practices, sometimes coming up against their own prejudices. At this point, the nurses’ and master’s students’ knowledge of Leininger’s theory served as a basis for team discussion and allowed them to reflect on the importance of culturally congruent care, so that the different types of knowledge (professional, personal and popular) must be weighed up in order to guarantee care that conforms to the cultural values of the group in question.

Thus, health education activities were developed with the aim of accommodating and restructuring cultural care in order to support, facilitate and enable the adjustment of habits/lifestyle in such a way as to be meaningful and congruent with the group being cared for. In this context, a health education action was developed in a playful way to address the issue of domestic hygiene with the families, without success in the perception of a change in habit. However, the team also invested in health education on oral hygiene with the children, using a dynamic approach, distributing oral hygiene kits, as well as constant guidance at each meeting between the team and the families and children. They were receptive, showing interest and giving positive feedback on subsequent visits.

With regard to the obstacles faced by the team, given that the Warao have their own dialect, the nurses and the rest of the team had to develop strategies for effective communication. Strategies such as mimicry, the use of images and approaching those who understood and spoke Portuguese were used to aid communication.

With regard to health care, the Warao people have their own beliefs, which involve the use of herbs and religious rituals, and these had to be respected by the health team. In this context, a conflict was experienced when an adolescent and his family refused to be admitted to hospital for treatment of tuberculosis with clinical complications. At this point, the team decided to preserve cultural care. However, the case worsened and the child had to be sent to the Intensive Care Unit (ICU). Even so, some Indigenous people went to the hospital and demanded that the patient be discharged. The situation was mediated through the accommodation and restructuring of cultural care, first evidenced by the team’s negotiation with the family and, later, with the hospital staff to allow the spiritual leader to enter the hospital to perform a healing ritual; a condition required to remain in the Intensive Care Unit bed for treatment.

Nursing and the interprofessional team faced the challenge of promoting health and preventing illnesses and diseases, but they also faced the dilemma of overcoming their own prejudices in order to assist users in a way that respected their culture. In this context, the knowledge and practical application of a nursing theory emerges as the fruit of this process, providing care that is more sensitive to the needs of others. This knowledge enables an understanding of the environment, the person, health and the nurse’s role in the face of demands, stimulating action with critical thinking.

## DISCUSSION

Madeleine Leininger’s theory on ethno-nursing or transcultural care argues that it is necessary to understand the socio-political-cultural context of an individual or certain group in order to understand their culture of care and its interactions with the popular (native to the local culture) and professional health systems (where nursing is usually primarily linked), so that nurses can establish three types of relationship with cultural care: preservation/maintenance, accommodation/adaptation, and restructuring/standardization^(14,17,18)^. Firstly, we will discuss the broader context of the Warao population.

The Warao people are the second largest ethnic group in Venezuela. They are based in the Orinoco River delta region and number approximately 50,000 people^(19)^. This area has been affected by oil exploration and the construction of dams, resulting in the salinization of the water and soil, impacting the activities that guarantee the livelihood of these people, such as fishing and agriculture^(20)^. In the 1990s, they faced the cholera epidemic, which decimated a significant number of this population. This context of economic crisis and loss prompted many of these Indigenous people to come to Brazil in 2014 and intensified in 2017. Thus, since 2018, the United Nations High Commissioner for Refugees (UNHCR), the United Nations (UN) and other organizations have turned to welcoming and protecting this group^(19,20)^.

As for the cities that received this immigration, the main ones were Boa Vista and Pacaraima, since they are municipalities that border Venezuela. Other cities with a significant number of this ethnic group are Manaus, Santarém and Belém. When they arrived in these places, the Warao lived on the streets, begging at traffic lights, staying in shelters or in rented houses or small collective rooms^(19,20)^. In the same way, the Warao arrived in Maceió. From February onwards, when the teams began to get closer and form bonds with these people, they found themselves on the streets and some in rented houses in precarious conditions, begging for food and donations to make ends meet.

When they arrived in the city of Boa Vista, the shelters housing the Warao were also overcrowded. This is a point that deserves attention, since these spaces are sometimes organized as emergency measures and staffed by professionals who are unaware of the culture of the Warao people and do not take it into account when sheltering them. In addition, this crowded environment compromises privacy, favors the spread of disease and violates the rights of these people^(19,21)^.

The emergency shelters in Maceio, which were organized in June due to the heavy rains, had an inadequate structure with crowding and a lack of privacy. As far as human resources are concerned, the fact that the Street Clinic team had already formed a bond with some Warao families and made regular visits to the shelters served to strengthen the relationship of trust, as well as being a bridge to inform the shelter staff of practices relevant to the culture of these people.

Thus, with regard to training human resources to work with cultural diversity, one study looks at the situation in Appalachia in the United States, which suffers from economic, cultural and health differences from the rest of the country, with the need to recruit professionals from outside the region or even from other countries, which contributes to the cultural disconnection between the professional and the person being cared for. The study points out that training professionals in the culture and beliefs of these people has improved communication and interaction between professional and patient (Appalachians)^(22)^.

In this way, nurses and nursing technicians as members of the Street Clinic team need to consider individualities in order to guarantee dignity in care. This approach contrasts with the biomedical model in which the patient is seen as dependent in the care process, without autonomy and participation in treatment. From a holistic perspective, the human being is seen in their singularity and diverse dimensions, which affects their experience during the health-disease-care process, where culture plays a role, as it characterizes the identity of a people, their values, symbols and norms, bringing belonging^(23)^.

Madeleine Leininger’s Transcultural Theory proposes the “sunrise” model to guide nurses in identifying human conditions in order to provide care. The model is made up of four levels: I) The cultural universe of each group, which must be grasped by the nurse in the exercise of care in order to interpret how their care will be received according to their traditions; II) The individual and family in the context of a health system in order to understand their meanings; III) Professional and popular knowledge in order to determine similarities and differences; IV) Nursing care that is coherent with cultures and valued by them^(14)^.

In this sense, in order to better understand the cultural universe and habits of the Warao people, the Street Clinic had to make an approach and form a bond. This is already part of the modus operandi of the Street Clinic, using soft technologies such as welcoming, listening and dialog to establish a relationship of trust with the user and enable comprehensive care, respecting the uniqueness of each person being cared for^(12)^. However, the communication barrier due to the Warao people’s dialect proved to be an obstacle to overcome. The experience of the Street Clinic in the state of Rio Grande do Norte also described the difficulty of communication as a limiting factor in the care process, since most of the Indigenous people speak the dialect of their own people and some speak Spanish, which has an impact on understanding the guidelines^(24)^.

Leininger’s Transcultural Care Theory understands care as a cultural act and that each people has its own particularities in this process. The theory was applied in the case of identifying habits that can be harmful to health, such as pans, plates and other utensils that are left on the floor, as well as how waste and garbage are dealt with. In addition to checking the children’s poor oral hygiene and the presence of pediculosis. Based on the theory, there was a process of getting to know the culture and trying to negotiate and restructure it by means of health education with frequent guidance that was taken up again at each visit, in order to sensitize them to the necessary domestic and hygiene care, but at this point, even with long-lasting assistance, it was not possible to observe any significant change in relation to this approach, which is very much linked to the customs of these people^(19,20)^. On the other hand, the health education practices with the children that took place, especially in October, were well received.

It’s worth noting that this health education experience confronted beliefs and challenged the nurses and the Street Clinic team to deal with their own prejudices. The way of dealing with hygiene made the team uncomfortable, but the experience required them to break with the biomedical model and look for strategies and references to better assist this population, valuing the integration between theory and practice, based on the understanding that needs are subjective and need to be listened to and (re)known in order to provide health care in nursing, respecting autonomy and cultural values.

Health education from the perspective of popular health education can be a powerful tool for achieving transcultural care, as it seeks to integrate the population’s knowledge with that of health professionals and value sociocultural aspects through listening and dialog, in order to build actions that strengthen people’s protagonism in health care practices^(25)^. Thus, it is necessary for health professionals to detach themselves in order to see reality through the user’s lens by building bonds, with a view to implementing comprehensive care^(25,26)^.

In addition, it was possible to observe the application of transcultural theory in the provision of care that is congruent with the values of the culture of the Warao people, in the case of the adolescent undergoing treatment for tuberculosis. The nurses and the health team tried to preserve the culture of health by respecting the family’s right to refuse hospitalization. However, as the patient’s condition worsened, there was a need to restructure the care offered in order to keep him in a better position to survive, and with the family’s agreement, the patient was hospitalized. After the group’s protest to maintain their health practices, it was necessary to negotiate with the social structure involved, and so the spiritual leader was allowed in to perform the ritual typical of this culture^(14,17,18)^.

In this specific case, it was possible to see the three ways in which the transcultural nursing approach can act: preservation – maintaining the family’s right to care for its member based on their customs; accommodation (negotiation) – to adjust the care already practiced by the Warao people to the hospital environment and the knowledge proposed by the team; and restructuring (repatterning) – intervening in the user’s hospitalization, changing the standard of care offered, seeking survival and restoration of health with tools typical of the professional health system, in this case, the SUS itself^(14,17,18)^.

A study of nurses from Yunnan province in China who provide transcultural care to ethnic minorities found that they also experienced challenges in nursing care and concerns about the quality of care provided. In order to improve and overcome these challenges and tensions, it was suggested that training should include points on transcultural knowledge and sensitivity, minority languages and multicultural experiences. Other facilitating strategies are suggested, such as a room with resources to instrumentalize health care and assistance, adaptation of religion and cuisine and a team to conduct transcultural care in hospitals^(27)^. As a result, the Street Clinic teams in Maceió are still looking for training and resources to better assist these people.

It’s worth considering that migration in Brazil continues and with this the patient population diversifies, added to the fact of the country’s own regional differences, nurses need to be sensitive to and knowledgeable about the culture in order to provide adequate care. To this end, transcultural care should be reinforced in the curricula from the time they graduate. A study in Slovenia of 318 first to third year nursing students at an educational institution found a satisfactorily high level of awareness of transcultural care among second year students, which demonstrates the successful application of the content and the importance of maintaining it throughout the course^(28)^.

A limitation of the study is that the data presented cannot be generalized, due to the characteristics of the methodology adopted and the theme addressed, since it presents the reality of nursing care provided to a foreign ethnic group in a particular region and this may vary depending on the region of Brazil, in addition to being a report by nurses, which may have different interpretations according to the experience and context in which each one works.

It can be inferred that this work will contribute to raising the awareness of professionals who deal with this public, encouraging them to deepen their studies based on ethno-nursing, seeking to understand more about the migratory phenomenon described, as well as ways in which nursing and public health can act, based on the planning of actions, strategies and perhaps health policies that are more specific to this population. With the trend towards economic and social union between Latin American countries, proposed by the current government forces, these migratory activities tend to become increasingly recurrent, which raises the need for new cross-cultural studies, such as the one presented here.

## CONCLUSION

The experience of nurses from the Street Clinic in caring for the Warao Indigenous population in the light of Madeleine Leininger’s Transcultural Theory favored significant social interaction and expanded the possibilities for achieving comprehensive healthcare. The process of providing care from an intercultural, intersubjective and interactional perspective challenged health professionals to break with their own prejudices and with an imposing model of health care, in search of cultural care that was consistent with the values of the Warao people. This theory guided the actions in such a way that it not only helped to restructure the habits of the Indigenous people, but also led to learning, change and reflection on the part of the team. Transcultural care recognizes the diversity of health care and efforts to integrate ethnically significant health practices with the professional health system.

Preserving the culture, Indigenous ancestral knowledge and beliefs of the Warao peoples involves nursing care, and for this it is necessary to acquire specific knowledge and skills, allowing these people the freedom to manage therapeutic resources and practices in dealing with illnesses. Without respect for sociocultural differences, positive interactions will become proportionally unlikely, as will the establishment of spaces for communication and mutual intelligibility between Indigenous Venezuelans and the professionals who care for them, missing out on opportunities for growth and learning on both sides in this process.

Deepening the transcultural study in nursing, based on Madeleine Leininger’s theory, in search of a better understanding of the culture of the Warao people, as well as their perspectives and care strategies, is fundamental in this new social configuration that Brazil is experiencing in various regions, currently concentrated in the North and Northeast. This does not exclude but encourages efforts by the humanities and health sciences to better understand this phenomenon, so that Brazilian institutions are prepared to manage and deal with this complex and dynamic process.
